# The epidemiology and outcomes of acute intestinal failure: a multicenter Argentine study

**DOI:** 10.62675/2965-2774.20260428

**Published:** 2026-06-03

**Authors:** Andrés Luciano Nicolás Martinuzzi, Eliana Quesada, Irina Aversa, Victoria Carolina González, Ezequiel Alfredo Manrique, Ailén Dietrich, Cayetano Galletti, Fernando Lipovestky, Sebastián Pablo Chapela

**Affiliations:** 1 Nutritional Support and Metabolism Committee Sociedad Argentina de Terapia Intensiva Buenos Aires Argentina Nutritional Support and Metabolism Committee, Sociedad Argentina de Terapia Intensiva - Buenos Aires, Argentina.; 2 Chapter of Graduates in Nutrition Sociedad Argentina de Terapia Intensiva Buenos Aires Argentina Chapter of Graduates in Nutrition, Sociedad Argentina de Terapia Intensiva - Buenos Aires, Argentina.

## INTRODUCTION

Acute intestinal failure (AIF) is a severe, life-threatening condition defined by the reduction of intestinal function below the minimum necessary, requiring intravenous supplementation.^(1,2)^ While European studies provide epidemiological benchmarks, reporting incidence rates of approximately 13 cases per 1,000 hospital admissions, the burden of AIF in Latin America remains largely unknown.^(3,4)^ This critical knowledge gap hinders the development of regional clinical guidelines, resource allocation for nutritional support, and the planning for long-term care, such as home parenteral nutrition (HPN).^(5)^ This study, the first of its kind in Argentina, aimed to determine the incidence, characteristics, and outcomes of AIF in the intensive care unit (ICU) setting.

## METHODS

We conducted a prospective, multicenter, observational study in six Argentinian ICUs from June 1st, 2023, to November 30, 2023. We included all adult patients (≥18 years) admitted to the ICU who were diagnosed with AIF and required parenteral nutrition (PN). Patients with pre-existing chronic intestinal failure were excluded. Acute intestinal failure was classified as type I (short-term, usually dysmotility-associated) or type II (prolonged, complex pathology) prospectively at the time of diagnosis by the local investigator, according to the European Society for Clinical Nutrition and Metabolism (ESPEN) criteria. The indication for PN followed strict ESPEN guidelines and the gastrointestinal failure score. Detailed inclusion criteria, definitions, and nutritional management protocols are provided in the Supplementary Material. The primary outcome was the incidence of AIF. Secondary outcomes included hospital length of stay (LOS), mortality, and discharge status (HPN dependency). Statistical analysis was performed using IBM Statistical Package for the Social Sciences (SPSS), version 24. Continuous variables were compared using Student's t-test or Mann-Whitney U test, and categorical variables using the Chi-squared test. A p value < 0.05 was considered significant. Institutional Ethics Committees approved the study.

## RESULTS

Among 2,704 ICU admissions during the study period, 81 patients developed AIF, yielding an incidence rate of 29.9 cases per 1,000 ICU admissions (95%CI 23.5 - 36.4). The cohort comprised 45 (55.6%) type I and 36 (44.4%) type II AIF patients (Figure 1S - Supplementary Material). As shown in table 1, type I patients were younger (p = 0.044) and had lower comorbidity scores (p = 0.043). Anthropometric characteristics (Table 1S), daily nutritional intake (Table 2S and Figure 2S), nutritional support type (Figure 3S), and PN-related complications (Table 3S) are detailed in the Supplementary Material. Conversely, type II patients, predominantly surgical, exhibited a more complex clinical course. Type II AIF patients had a significantly longer hospital LOS (median 30.5 *versus* 23 days, p = 0.048). Although overall mortality (24.7%) did not differ statistically between groups (p = 0.565), a critical difference was observed at discharge. While 0% of type I patients required HPN, 19.4% (7/36) of type II AIF patients were discharged requiring HPN (p = 0.004).

**Table 1:** Baseline characteristics

		AIF n = 81	AIF type I n = 45	AIF type II n = 36	p value
Age (years)		60.0 ± 18.7	56.3 ± 19.0	64.7 ± 17.5	0.044
Gender M: F		45 (55.6):36 (44.4)	28 (62.2):17 (37.8)	17 (47.2):19 (52.8)	0.177
NRS 2002		3.85 ± 1.35	3.73 ± 1.4	4.0 ± 1.2	0.381
SGA					
	A (well nourished)	22 (27.2)	17 (37.8)	5 (13.9)	
	B (moderately malnourished)	35 (43.2)	18 (40.0)	17 (47.2)	0.042
	C (severely malnourished)	24 (29.6)	10 (22.2)	14 (38.9)	
Charlson Comorbidity Index		3 (1 - 5)	2 (1 - 4)	4 (2 - 6)	0.043
APACHE II		11 (8 - 17)	13 (8 - 19)	10 (7 - 14.5)	0.225
Patient type					
	Medical	40 (49.8)	25 (55.5)	15 (36.5)	
	Surgical	41 (50.6)	20 (44.5)	21 (63.5)	0.091
Oncologic active disease		38 (46.9)	19 (42.2)	19 (52.8)	0.344
Pathophysiological classification					
	SBS	5 (6.2)	-	5 (13.8)	
	Intestinal fistula	6 (7.4)	-	6 (16.7)	
	Mechanical obstruction	19 (23.5)	-	19 (52.9)	
	Intestinal dysmotility	45 (55.6)	45 (100)	-	
	ESBMD	5 (6.2)	-	5 (13.8)	N/A
	CIPO	1 (1.2)	-	1 (2.8)	
PN formula					
	Tailor made	11 (13.6)	3 (6.7)	8 (22.2)	0.042
	Ready-to-use	70 (86.4)	42 (93.3)	28 (77.8)	
PN (days)		12 (7 - 21)	9 (6.5 - 14)	19 (10 - 36.7)	
ICU LOS		11 (5.5 - 22)	10 (5 - 19.5)	14 (7 - 29.7)	0.499
Hospital LOS		25 (17 - 41)	23 (14 - 30.5)	30.5 (18.5 - 47.5)	0.048
Outcome					
	Discharged	54 (66.7)	35 (77.8)	19 (52.8)	0.565
	Discharge with HPN	7 (8.6)	-	7 (19.4)	0.000
	Death	20 (24.7)	10 (22.2)	10 (27.8)	0.565

AIF - acute intestinal failure; M - male; F - female; NRS - nutritional risk screening; SGA - subjective global assessment; APACHE II - Acute Physiology and Chronic Health Evaluation; SBS - short bowel syndrome; ESBMD - extensive small bowel mucosal disease; CIPO - chronic idiopathic intestinal pseudo-obstruction; PN - parenteral nutrition; ICU - intensive care unit; LOS - length of stay; HPN - home parenteral nutrition. Results expressed in mean ± standard deviation; median (interquartile range) or n (%).

## DISCUSSION

This is the first multicenter study to establish the epidemiological burden of AIF in Argentinian ICUs. Our finding of 29.9 cases per 1,000 admissions provides a critical benchmark for resource planning. This incidence is higher than the 13 cases per 1,000 hospital admissions reported in Europe, reflecting our specific focus on a high-acuity ICU population and active screening by nutritional support teams.^(3)^ In line with international literature, the primary causes in our cohort were gastrointestinal dysmotility (type I) and complex abdominal pathology (type II).^(3)^ The most significant clinical finding is the high rate of progression to chronic intestinal failure; nearly one in five type II AIF patients required HPN at discharge.^(6)^ This figure highlights a substantial long-term healthcare burden and underscores the urgent need for structured intestinal rehabilitation and transitional care programs.^(7-10)^

## DECLARATION OF GENERATIVE AI & AI-ASSISTED TECHNOLOGIES IN THE WRITING PROCESS

Artificial Intelligence (AI) tools, specifically ChatGPT (OpenAI, GPT-4), were used to improve the clarity of English grammar, spelling, and language structure in the preparation of this manuscript. These tools were employed to enhance the readability and presentation of the text but were not used to generate scientific content, interpret data, or influence the study's conclusions. All intellectual and scientific contributions, as well as the accuracy and integrity of the data presented, remain the sole responsibility of the authors. The final manuscript was thoroughly reviewed and approved by the authors to ensure compliance with ethical and professional standards for scientific publication.

## Data Availability

The datasets used and/or analyzed during the current study are available from the corresponding author on reasonable request.
