# Supplementary material for: The epidemiology and outcomes of acute intestinal failure: a multicenter Argentine study
Source: Crit Care Sci. 2026 Jun 3;38:e20260428. doi: 10.62675/2965-2774.20260428 (PMC13399227; doi:10.62675/2965-2774.20260428)
Supplement: Supplementary Material [file 2965-2774-ccsci-38-e20260428-Suppl01.pdf]

Critical Care Science
https://doi.org/10.62675/2965-2774.20260428

SUPPLEMENTARY MATERIAL

The epidemiology and outcomes of acute intestinal failure: a multicenter Argentine study

Andrés Luciano Nicolás Martinuzzi[1], Eliana Quesada[2], Irina Aversa[1], Victoria Carolina González[1], Ezequiel Alfredo Manrique[1], Ailén Dietrich[2], Cayetano Galletti[1], Fernando Lipovestky[1], Sebastián Pablo Chapela[1]

1. SUPPLEMENTARY METHODS

Study design and settings

This was a prospective multicenter observational study. The enrolment period for centers extended from February 2023 to May 2023. Six centers from different regions of Argentina participated. Patient registration occurred from June 1st, 2023, to November 30, 2023.

Inclusion and exclusion criteria

We included patients of both sexes, aged ≥ 18 years, admitted to the intensive care unit (ICU) diagnosed with acute intestinal failure (AIF) requiring parenteral nutrition (PN). Exclusion criteria were age < 18 years, therapeutic effort limitations or palliative care, pre-existing chronic intestinal failure upon admission, and pregnancy.

Definitions

PN: the administration of macronutrients (glucose, amino acids, and lipids) via intravenous access. Administration of fluids or electrolytes alone was not considered PN.

AIF: defined based on European Society for Clinical Nutrition and Metabolism (ESPEN) recommendations as the reduction of intestinal function below the minimum necessary for the absorption of macronutrients and/or water and electrolytes. thereby requiring intravenous supplementation (IVS).

AIF classification: Patients were classified prospectively by the local principal investigator at the time of PN indication:

- Type I AIF: acute. short-term condition (e.g., dysmotility in critical illness, postoperative ileus).
- Type II AIF: prolonged acute condition in metabolically unstable patients. typically associated with abdominal catastrophes (e.g., ischemia, fistulas) requiring complex multidisciplinary management.
- Note: the distinction was based on the clinical nature of the underlying pathology and ESPEN criteria, not on a retrospective day-count threshold.

Home PN (HPN): defined as the requirement for PN in patients who were medically stable for discharge and were effectively discharged to their homes with an established HPN regimen.[1]

Nutritional management and PN indication: Indication for PN followed the recommendations of the Nutritional Support and Metabolism Committee (COSONUME) and ESPEN guidelines.

This is an open access article under the CC BY license (https://creativecommons.org/licenses/by/4.0/).

Gastrointestinal failure (GIF) score was used to guide decision-making. Parenteral nutrition was indicated when enteral nutrition (EN) was not feasible for > 3 days, or in cases of severe feeding intolerance/contraindication (GIF Score 3 - 4), ensuring that PN was not used liberally but as a rescue therapy for intestinal failure.

Sample size calculation

The sample size was determined *a priori* to ensure statistical validity for the primary outcome (prevalence of AIF). We used the formula:

$$N = \frac{Z^2 * P(1-P)}{E^2}$$

In which:

– Z (confidence level) = 1.96 (for 95% confidence).

– P (expected prevalence of AIF among ICU admissions) = 4.8% (0.048), based on a prior pilot study in Argentinian critical care units.[(2)]

– E (Desired precision/absolute margin of error) = 1% (0.01).

The calculated minimum sample size of ICU admissions was 1.756 patients. The final enrolled cohort consisted of 2.704 ICU admissions (yielding 81 AIF cases), which satisfies this requirement and provides high statistical power for the incidence estimation. This patient's flowchart is represented in figure 1S.

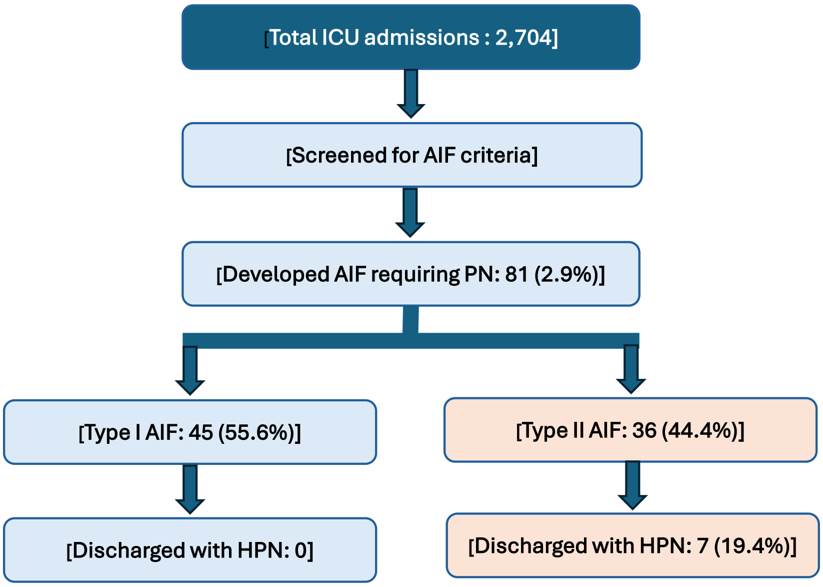

ICU - intensive care unit; AIF - acute intestinal failure; PN - parenteral nutrition; HPN - home parenteral nutrition.
Figure 1S - Patient flowchart.

2. SUPPLEMENTARY RESULTS

Patients anthropometric characteristics

Mean current and regular body weight, height and body mass index were registered upon admission. As shown in table 1S there were no significant difference on those variables within patients with type I and II AIF.

Table 1S - Patients anthropometric characteristics

	AIF n = 81	AIF type I n = 45	AIF type II n = 36	p value
Current weight (kg)	76.1 ± 21.6	76.6 ± 18.9	76.8 ± 24.7	0.976
Regular weight (kg)	80.9 ± 20.1	80.8 ± 19.5	80.9 ± 21.1	0.987
Height (cm)	169.1 ± 9.1	169.8 ± 9.1	168.4 ± 9	0.618
BMI (kg/cm^2)	26.7 ± 6.4	26.5 ± 5.3	27.1 ± 7.6	0.193

AIF - acute intestinal failure; BMI - body mass index.

Nutritional intake

Mean caloric and protein intake were monitored daily. As shown in table 2S and figure 2S. Patients with Type II AIF had significantly higher protein intake during the first three days of nutritional support (Day 0: p = 0.003; Day 1: p = 0.033; Day 2: p = 0.042) than type I patients. This was likely driven by the increased use of supplemental PN (SPN) combined with EN in the type II group, as illustrated in figure 3S.

Table 2S - Mean total calories and protein intake (first 14 days)

		AIF			AIF		
		Total calories			Total proteins		
		Type I	Type II	p value	Type I	Type II	p value
		Mean ± SD	Mean ± SD		Mean ± SD	Mean ± SD	
NS day	0	661.9 ± 236.6	534.4 ± 305.7	0.037	24.8 ± 11.6	33.2 ± 11.2	0.003
	1	671.5 ± 415.1	824 ± 424.6	0.146	31.9 ± 11.3	37.9 ± 14.6	0.033
	2	1,085.1 ± 471.5	1,285.6 ± 501.1	0.066	50.2 ± 22.6	60.8 ± 26	0.042
	3	1,378.2 ± 542.6	1,499.1 ± 560.8	0.284	65.4 ± 28.6	69.9 ± 26.3	0.412
	4	1,557.7 ± 530.4	1,681.2 ± 447	0.269	73.5 ± 28.8	82.8 ± 30	0.125
	5	1,518.2 ± 604.3	1,657.6 ± 472.6	0.261	71.5 ± 31.2	76.9 ± 22.1	0.371
	6	1,584.6 ± 626.2	1,714.9 ± 506.8	0.318	74.5 ± 31.6	84.2 ± 32.6	0.129
	7	1,688.4 ± 579.8	1,724.2 ± 457.2	0.769	81.3 ± 33.3	87.5 ± 32.6	0.403
	8	1,667.4 ± 633.8	1,736.7 ± 605.7	0.639	77.9 ± 34.1	88.7 ± 38.7	0.169
	9	1,749.4 ± 523.6	1,827.7 ± 501.6	0.498	85.4 ± 28.1	96.3 ± 40.6	0.15
	10	1,681.9 ± 575.4	1,785.5 ± 515.3	0.399	82.5 ± 32.9	93.1 ± 39.7	0.167
	11	1,568.5 ± 629.8	1,819.2 ± 486.3	0.055	76.8 ± 37.5	93.8 ± 38.5	0.048
	12	1,,528.6 ± 689.8	1,755.3 ± 632.2	0.135	72.5 ± 30.1	93.5 ± 44.3	0.001
	13	1789.9 ± 545.9	1,871.2 ± 558.1	0.507	88.5 ± 30.6	99.7 ± 40.9	0.162
	14	1,562.8 ± 580.9	1,793.3 ± 508.5	0.063	78.1 ± 35.6	92.7 ± 35.8	0.076

AIF - acute intestinal failure; NS - nutrition support; SD - standard deviation.

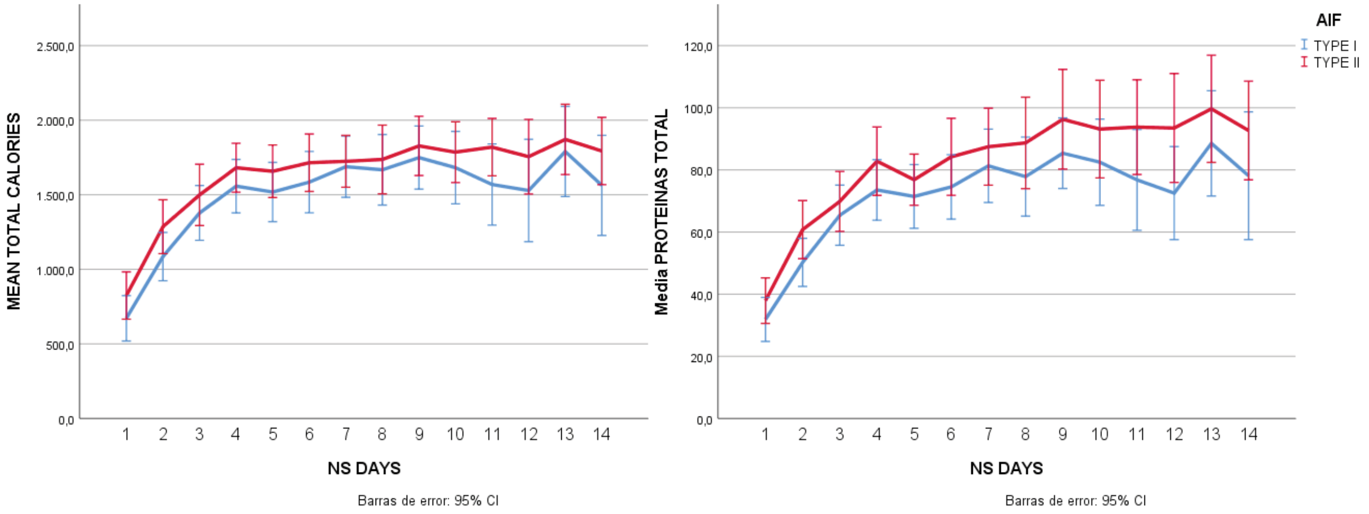

AIF - acute intestinal failure; NS - nutrition support.

Figure 2S - Mean total calories and protein intake.

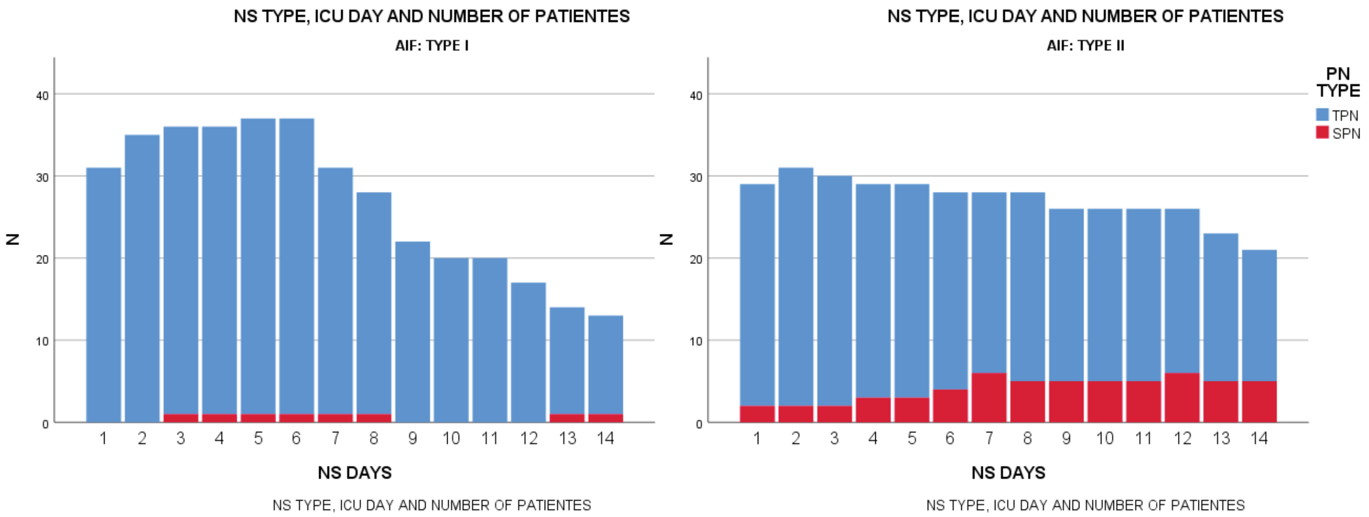

AIF - acute intestinal failure; PN - parenteral nutrition; TPN - total parenteral nutrition; SPN - supplemental parenteral nutrition; NS - nutrition support.

Figure 3S - Nutritional support type (total parenteral nutrition versus supplemental parenteral nutrition) by day.

COMPLICATIONS

Incidence of PN-related complications is detailed in table 3S. There were no significant differences in metabolic complications or catheter-related bloodstream infections (CLABSI) between groups.

Table 3S - Parenteral nutrition-related complications

	All AIF n = 81	AFI type I n = 45	AIF type II n = 36	p value
Catheter related				
CLABSI	4 (4.9)	1 (2.2)	3 (8.3)	0.207
CLABSI rate (1,000 CVC days)	3.81	2.02	4.98	0.463
CRVT	-	-	-	
Catheter replacement events	0 (0 - 1)	0 (0 - 1)	0 (0-2)	0.044
Metabolic				
Hyperglycaemia	21 (25.9)	12 (26.7)	9 (25.0)	0.865
Hypertriglyceridemia	11 (13.5)	7 (15.6)	4 (11.1)	0.562
AST and/or ALT elevation	19 (23.4)	4 (31.1)	5 (13.9)	0.069
Hyperbilirubinemia	14 (17.3)	9 (20.0)	5 (13.9)	0.470

AIF - acute intestinal failure; CLABSI - central line-associated bloodstream infection; CVC - central venous catheter; CRVT - central venous catheter-related thrombosis; AST - aspartate aminotransferase; ALT - alanine aminotransferase. Results expressed as n (%) or median (interquartile range).

To provide context with the current literature:

- **Infectious complications**: our overall CLABSI rate (3.81 per 1,000 catheter days) was comparable to the 3.63 per 1.000 catheter days reported by Reintam Blaser et al. in the largest international multicenter study on AIF,[3] reflecting the high standards of catheter care in the participating centers.
- **Metabolic complications**: the observed rates of hyperglycemia (25.9%) and liver dysfunction (23.4%) are consistent with the severity of illness described in previous AIF cohorts.[3] These findings underscore the profound metabolic stress and inflammatory burden associated with AIF in the ICU setting.

However, catheter replacement events were significantly more frequent in type II AIF patients (p = 0.044), reflecting the longer duration of therapy and vascular access challenges in this population.

REFERENCES

1. Cuerda C. Pironi L. Arends J. Bozzetti F. Gillanders L. Jeppesen PB. et al.; Home Artificial Nutrition & Chronic Intestinal Failure Special Interest Group of ESPEN. ESPEN practical guideline: clinical nutrition in chronic intestinal failure. Clin Nutr. 2021;40(9):5196-220.
2. Martinuzzi AL. Manrique E. Roel P. Cornú M. Lombi Y. Sobre la incidencia de la falla intestinal aguda en las unidades de cuidados críticos de la Argentina. Rev Cubana Aliment Nutr. 2021;31(1):1-32.
3. Reintam Blaser A. Ploegmakers I. Benoit M. Holst M. Rasmussen HH. Burgos R. et al.; AIF study group. Acute intestinal failure: international multicenter point-of-prevalence study. Clin Nutr. 2020;39(1):151-8.
